# Metformin treatment reduces motor and neuropsychiatric phenotypes in the zQ175 mouse model of Huntington disease

**DOI:** 10.1038/s12276-019-0264-9

**Published:** 2019-06-05

**Authors:** Ana Sanchis, María Adelaida García-Gimeno, Antonio José Cañada-Martínez, María Dolores Sequedo, José María Millán, Pascual Sanz, Rafael P. Vázquez-Manrique

**Affiliations:** 1 Research Group in Molecular, Cellular and Genomic Biomedicine, Health Research Institute La Fe (Hospital Universitario y Politécnico La Fe), València, Spain; 2 Department of Biotechnology, Escuela Técnica Superior de Ingeniería Agronómica y del Medio Natural (ETSIAMN), Universitat Politécnica de València, València, Spain; 3 Statistics Unit, Health Research Institute La Fe (Hospital Universitario y Politécnico La Fe), València, Spain; 4 CIBER de Enfermedades Raras (CIBERER), Madrid, Spain; 5 Instituto de Biomedicina de València, CSIC, València, Spain

**Keywords:** Huntington's disease, Molecular neuroscience

## Abstract

Huntington disease is a neurodegenerative condition for which there is no cure to date. Activation of AMP-activated protein kinase has previously been shown to be beneficial in in vitro and in vivo models of Huntington’s disease. Moreover, a recent cross-sectional study demonstrated that treatment with metformin, a well-known activator of this enzyme, is associated with better cognitive scores in patients with this disease. We performed a preclinical study using metformin to treat phenotypes of the zQ175 mouse model of Huntington disease. We evaluated behavior (motor and neuropsychiatric function) and molecular phenotypes (aggregation of mutant huntingtin, levels of brain-derived neurotrophic factor, neuronal inflammation, etc.). We also used two models of polyglutamine toxicity in *Caenorhabditis elegans* to further explore potential mechanisms of metformin action. Our results provide strong evidence that metformin alleviates motor and neuropsychiatric phenotypes in zQ175 mice. Moreover, metformin intake reduces the number of nuclear aggregates of mutant huntingtin in the striatum. The expression of brain-derived neurotrophic factor, which is reduced in mutant animals, is partially restored in metformin-treated mice, and glial activation in mutant mice is reduced in metformin-treated animals. In addition, using worm models of polyglutamine toxicity, we demonstrate that metformin reduces polyglutamine aggregates and restores neuronal function through mechanisms involving AMP-activated protein kinase and lysosomal function. Our data indicate that metformin alleviates the progression of the disease and further supports AMP-activated protein kinase as a druggable target against Huntington’s disease.

## Huntington’s disease: Diabetes drug limits disease-related degeneration in mice

Metformin, an existing drug for diabetes, shows promise in alleviating symptoms of early Huntington’s disease in mouse models. Huntington’s disease is a genetic disorder that results in the gradual deterioration of motor skills and cognitive ability. It is caused by a defect in a single gene that then encodes a mutant huntingtin protein, which aggregates and kills brain cells. Growing observational evidence suggests that patients undergoing metformin treatment for diabetes type II exhibit fewer symptoms of age-related disease, as well as Huntington’s disease. Rafael Vázquez-Manrique at Hospital Universitario y Politécnico La Fe, València and Pascual Sanz at IBV-CSIC and CIBERER, València, and scientists across Spain used metformin to treat motor and neuropsychiatric symptoms in a Huntington’s mouse model. They found that metformin alleviated symptoms by actively reducing huntingtin levels, dispersing aggregations and limiting brain inflammation.

## Introduction

Huntington disease (HD) is a dominant, inherited neurodegenerative disorder that leads to impaired motor coordination associated with chorea and progressive deterioration of cognitive function. Patients with HD have an abnormal CAG expansion within the first exon of the huntingtin gene, *HTT*. This gene encodes a cytosolic protein, huntingtin (Htt), the function of which is unclear. When *HTT* has 35 or more CAG triplet repeats, the protein contains abnormally long glutamine tracts (polyQ), resulting in a mutant huntingtin (mHtt), which shows toxic gain-of-function properties. mHtt is prone to improper folding and to forming aggregates, thereby perturbing a range of essential cellular functions^1^ and impairing cell viability with particularly severe effects in neurons of the striatum^2^.

Neurons respond to toxic mHtt by activating pathways of protein clearance, such as autophagy^3^ or the proteasome^4^. Among the molecules that can induce autophagy, AMP-activated protein kinase (AMPK), the master regulator of energy homeostasis in eukaryotic cells, plays a key role^5,6^. This enzyme is a heterotrimer (AMPKα is catalytic and AMPKβ and AMPKγ are regulatory subunits^7,8^) that is activated when ATP levels are reduced. Activated AMPK coordinates metabolism, cell growth, and autophagy, and through the autophagy pathway, AMPK activation is able to reduce the levels of mHtt^3^. Activation of AMPK by genetic and pharmacological means also reduces neuronal polyQ-induced toxicity in *Caenorhabditis elegans*^9^ and reduces cell death in a mammalian in vitro model of HD^9^, which is accompanied by a reduction in mHtt aggregates. Moreover, overexpression of the AMPKγ gain-of-function mutant reduces neuronal loss in a mouse model of HD^9^. Therefore, this enzyme has been proposed as a druggable target against HD^9^.

Reducing mHtt expression in models of HD reverses phenotypes associated with the disease^10,11^. One potential substance that may induce mHtt lowering is metformin, a well-known AMPK activator^12,13^. Metformin is used worldwide to treat type 2 diabetes mellitus^14^. Interestingly, researchers discovered that patients suffering from this disease who are chronically taking metformin experience unexpected side benefits, such as protection against cancer^15^ and nephrotic symptoms^16^, among others. Metformin demonstrates cell protection properties in polyQ-expressing *C. elegans* and in vitro models of HD^9^. In addition, metformin treatment also increases lifespan, among other effects, in males of the R6/2 mouse model of HD^17^. Finally, a statistical analysis of participants in the Enroll-HD database, a worldwide observational study on HD, showed that metformin intake is associated with better cognitive function in HD patients^18^, strongly pointing to metformin as a putative treatment for HD.

In this study, *C. elegans* was used to illustrate that metformin is able to reduce the aggregation of polyQs and neuronal impairment in worms in an AMPK- and lysosomal-dependent manner. We also performed a protocol to test the beneficial effects of metformin in the zQ175 mouse model of HD at 3 months of age (i.e., early stages of HD). After 3 months of treatment, untreated heterozygous zQ175 animals showed strong neuropsychiatric and motor defects that were partially prevented by metformin treatment. In the same way, untreated zQ175 animals exhibited morphological abnormalities in the striatal neurons that were ameliorated in animals treated with metformin. Moreover, in metformin-treated mice, mHtt aggregation was reduced, and brain-derived neurotrophic factor (BDNF) levels, which were lower in untreated HD mice than in controls, showed a clear recovery. Analysis of the cortex of these animals showed that metformin treatment was also able to ameliorate the brain inflammatory response in zQ175 animals.

## Materials and methods

### Worm culture and transgenesis

Worms were cultured using techniques and media described elsewhere^19^. The genotype of the *C. elegans* strains used in this work is described in Table 1. Worms were cultured at 20 °C. The 112Q worm model was created by microinjection of a mixture of plasmids: 5 ng/μl of pCFJ90 (final concentration), 25 ng/μl of pRVM-112Q, and 100 ng/μl of pYES2 as a carrier DNA, as described elsewhere^20,21^. pRVM-112Q was created using the Gateway system (Invitrogen, Waltham, MA, USA), fusing three components, the promoter of the *mec-3* gene to drive the expression of the construct in mechanosensory neurons, 112CAG::TdTomato, and the terminator of transcription of the *unc-54* gene into the destination vector pCFJ150. 112CAG::TdTomato was synthesized de novo in General Biosystems (Morrisville, NC, USA). This construct was codon optimized for *C. elegans* with three artificial introns to facilitate expression. The sequence of 112CAG::TdTomato was flanked by the attB1 and attB2 sequences to allow recombination in pDONR221 vectors (pDONR-P4-P1r-*mec-3p* and pDONR-P2r-P3-*unc-54*T). pCFJ90 (*Pmyo-2::mCherry::unc-54utr*; Addgene plasmid # 19329) and pCFJ150 (pDESTttTi5605 [R4-R3]; Addgene plasmid # 19327) were gifts from Erik Jorgensen^22^ (Additional file 1: Fig. S1).

**Table 1:** *Caenorhabditis elegans* strains used in this work

Strain^a^	Genotype	Reference
Bristol N2	Standard wild type	^61^
RB754	*aak-2(ok524)* X	^62^
AM141	*rmIs133 [unc-54p::Q40::YFP]*	^32^
RVM137	*rmIs133 [unc-54p::Q40::YFP]; aak-2(ok524)X*	This work
RVM131	*vltEx131*[*mec-3p::112Q::TdTomato; myo-2p::GFP*]	This work
RVM132	*vltEx132*[*mec-3p::112Q::TdTomato; myo-2p::GFP*]; *aak-2(ok524)* X;	This work

^a^All mutant strains were out-crossed at least three times. N2, CF1038, and RB754 were provided by the Caenorhabditis Genetics Center (University of Minnesota, MN, USA)

### Phenotypical assays on worms

Touch assays were performed by gently passing an eyelash, mounted on a toothpick, through the tail of the worms, as described elsewhere^23^. The tail of each worm was scored approximately 10 times. This test was performed on approximately 50 animals, and the average response was plotted. Each assay was repeated at least three times.

To estimate the rate of aggregation of polyQ-containing proteins, the number of inclusion bodies was counted in 40Q::YFP animals using a dissecting microscope equipped with fluorescence (Leica M165FC; Leica, Wetzlar, Germany). At least 30 animals of each strain and/or treatment group were counted.

### Drug assays on worms

Worms used for touch assays were grown in 96-well plates with liquid medium to a final volume of 50 μl. Animals used to estimate the number of inclusion bodies were grown in 50 ml tubes to a final volume of 5 ml. Both liquid cultures consisted of a mix of M9, *Escherichia coli* as a source of food (strain OP50; used a proportion of 1:9 of a suspension with an OD600 = 0.5), 50 mg/ml streptomycin, 12.5 mg/ml nystatin, and the corresponding amount of metformin/vehicle. Twenty-four hours before the assays were performed, 10 µM chloroquine was added to the medium. Metformin was used at a concentration of 2 mM, unless otherwise stated. Both drugs were obtained from Sigma-Aldrich (St. Louis, MO, USA). Images of polyQ worms were taken using a confocal microscope (Leica TCS SP5 Confocal microscope, Leica Microsystems SLU, Barcelona, Spain) belonging to the Microscopy Unit of the IIS-La Fe (Valencia, Spain), as described before^24,25^.

### Mouse maintenance, treatment, and analysis

This study was carried out in strict accordance with the recommendations in the Guide for the Care and Use of Laboratory Animals of the Consejo Superior de Investigaciones Cientificas (CSIC, Spain). All mouse procedures were approved by the animal committee of the Instituto de Biomedicina de Valencia-CSIC [Permit Number: IBV-18 and IBV-22]. All efforts were made to minimize animal suffering. Male mice were sacrificed by cervical dislocation. The brain was recovered, and the two hemispheres were separated: one was conserved at −80 °C for molecular analysis, and the other was fixed in 4% paraformaldehyde in phosphate-buffered saline (PBS) for immunohistochemical analyses.

Mice were group housed (4–5/cage) in standard cages with wood shavings, and the environment was enriched with shredded paper. Breeder animals also received cotton nestles. Food and water were available ad libitum. Temperature (22–26 °C) and humidity (45-65%) were controlled and monitored daily, and a 12 h light–dark cycle (7:00 a.m.–19:00 p.m.) was instituted. Mice were housed at the Instituto de Biomedicina de Valencia-CSIC (València, Spain).

The zQ175 mouse, provided by the CHDI Foundation, carries a normal murine *Htt* allele and a knock-in (KI) mutant *Htt/HTT* mouse/human hybrid allele containing approximately 190 CAG repeats. Heterozygous and wild-type (WT) mice were generated by crossing heterozygous zQ175 mice in a C57BL/6J background. The genotype of the offspring was verified by PCR using genomic DNA extracted from tail tips. Biochemical and behavioral experiments were performed using littermates from the same population, which were randomly assigned to the treated or untreated groups. Only male mice were used in this study as a first approach to determine whether metformin treatment is able to ameliorate the HD phenotypes.

To genotype the CAG expansions of the mice, we obtained genomic DNA from tail and cortex tissues using the DNeasy Blood & Tissue Kit (Qiagen, Venlo, The Netherlands). PCRs were performed to amplify the expansion of CAGs using the following primers synthesized in IDTDNA (Coralville, IA, USA): HD-large-Forward (marked with 6-carboxyfluorescein [6- FAM] 5ʹ-ATG GCG ACC CTG GAA AAG CTG ATG AA-3ʹ and HD-large-Reverse 5ʹ-GGC GGC TGA GGA AGC TGA GGA-3ʹ, using the following program: 95 °C for 5 min and then 35 cycles of 95 °C for 45 s, 67 °C for 1 min, and 30 s and 72 °C for 5 min; finally, an extension step was performed at 72 °C for 10 min FAM-labeled PCR products were analyzed in an ABI PRISM 3100 Genetic Analyzer (3130xl System Upgrade) (Thermo Fisher, Waltham, MA, USA), together with the GeneScan 600 LIZ dye Size Standard v2.0 (Thermo Fisher), using the GeneMapper software (Thermo Fisher). We assumed that the mean number of CAG triplets of the mutant allele corresponded to the peak with the highest intensity in the center of the normal distribution formed by the PCR fragments. The amplified CAG repeat was flanked by 100 bp, which includes the primer sequences. Therefore, the mean number of CAG triplets of each sample was obtained using the following formula: average number of CAG triplets = (bp of the highest peak − 100)/3. We present the raw data results in Supplementary Fig. S4 (tail samples) and S5 (cortex samples). The comparison of the CAG expansions is shown in Supplementary Table S1 and shows no substantial dispersion among the mouse groups.

Three-month-old heterozygous zQ175 male mice were treated with 2 mg/ml metformin, administered ad libitum with the drinking water, for 3 months. This dose was chosen because the optimal oral metformin dose for many diabetic patients (approximately 20 mg/kg/day) has been translated into the mouse equivalent dose of 250 mg/kg/day^26^.

A battery of behavioral tests at the beginning and end of the treatment (Additional file 1: Fig. S2) was performed. During this period, no differences in body weight or other health parameters were observed among treatment groups and control animals (Additional file 1: Fig. S3). A total of 29 heterozygous male zQ175 mice (HD mice from now) and 22 WT mice were used in this study, distributed as follows: 12 HD and 8 WT mice received metformin treatment, and 17 HD and 14 WT mice were left untreated.

### Mouse behavioral testing

The behavior assessment was initiated when animals were 3 months old (pretreatment) and then repeated after 3 months of treatment (6 months old; post treatment). We consider this interval of time, from 3 to 6 months of age of the zQ175 animals, as the early phases of HD. Experiments were performed with at least 1–2 day intervals between tests. The results were scored by a person blinded to genotype and treatment.

#### Tail suspension test

Animals were suspended by their tails, and the amount of “immobility” time was measured^27^. A mouse was judged to be immobile when it hung by its tail without engaging in any active behavior. Test sessions lasted for 6 min, and scoring was performed by a single experienced observer who was blinded to the genotypes and treatments. Latency to immobility and duration of immobility were measured.

#### Beam balance

Beam balance assesses a mouse’s ability to maintain balance while traversing a narrow beam to reach a safe platform^28^. Three different beams are used in the assay with widths of 30, 12, and 5 mm. Decreasing the width of the path increases the fear associated with crossing the beam and impairs animal performance. This assay is used for the early detection of motor deficits in mouse models of HD. The recorded measurements included the time taken to cross the beam and the number of paw faults or slips.

#### Rotarod

The rotarod was specifically designed for making automated measurements of neurological deficits in rodents and is one of the most commonly used tests of motor function in mice^28^. Mice were tested over 3 consecutive days. Each daily session included a single training trial of 5 min at 4 rpm on the rotarod apparatus. One hour later, the animals were tested for three consecutive accelerating trials of 5 min with the rotarod speed changing from 0 to 40 rpm over 300 s, with an intertrial interval of at least 30 min The latency to fall from the rod was recorded for each trial. Mice that remained on the rod for more than 300 s were removed, and their latency was scored at 300 s.

### Immunohistochemistry

Dehydrated tissues from at least three independent mice per group were embedded in paraffin, and sagittal brain slices were sectioned at 5 μm. Sections corresponding to Fig. 107 of the Paxinus and Franklin’s mouse brain atlas were dewaxed, rehydrated, and warmed at 95 °C for 20 min in 10 mM citrate buffer for antigen retrieval. Sections were blocked in blocking buffer (1% bovine serum albumin; 5% fetal bovine serum in PBS) and incubated overnight at 4 °C with the appropriate primary antibody diluted in blocking buffer: anti-glial fibrillary acidic protein (GFAP) (diluted 1/600; Sigma ref. #G3893), anti-ionized calcium-binding adaptor molecule 1 (IBA1) (diluted 1/200; Wako ref. #019-19741), anti-brain-BDNF (diluted 1/500; Santa Cruz ref. #sc-546), anti-mHtt mEM48 (diluted 1/100; Merck Millipore ref. #MAB5374), anti-p62 (diluted 1/500; Abcam ref. #ab56416), anti-phosphorylated acetyl CoA carboxylase (pACC) (diluted 1/100; Cell Signalling ref. #3661), and anti-phospho-Thr202/Tyr204-extracellular signal-regulated kinase1/2 (pERK1/2) (diluted 1/400; Cell Signalling ref. #4376). After three washes of 10 min in PBS, sections were incubated for 1 h at room temperature with the corresponding biotin-conjugated anti-rabbit or anti-mouse secondary antibody (Jackson ImmunoResearch, West Grove, PA, USA) diluted in blocking buffer, washed three times with PBS for 5 min, and visualized with Avidin–Biotin Complex (ABC) (Vectastain Elite ABC Kit, Vector Laboratories, Burlingame, CA, USA) using diaminobenzidine as the chromogenic substrate for peroxidase (Peroxidase Substrate Kit DAB; Vector Laboratories, Burlingame, CA, USA). Sections were slightly counterstained with hematoxylin (Sigma, Madrid, Spain), dehydrated, and mounted in DPX (Merck, Germany). Images were acquired by light microscopy (Leica DM RXA2, Leica Microsystems, Wetzlar, Germany) and analyzed with the ImageJ software (NIH, Bethesda, MD, USA). To quantify the diaminobenzidine (DAB) intensity of each sample, we used a macro for ImageJ developed for automatic DAB stain detection and semiquantitative analysis^29^. This macro included a background subtraction algorithm followed by color deconvolution calibrated to DAB and hematoxylin stains in individual stained slides^30^. Comparison of different parameter combinations was achieved by overlaying the measured area to the original picture^31^. Before final data collection, the macro was refined and validated in a randomly chosen subset of pictures for each experimental group. Then, the amount of positive staining is expressed as the percentage area of positive staining relative to the total area of the picture. Experiments were scored by a person blinded to genotype and treatment.

### Western blot analyses

Mouse brain homogenates were lysed in RIPA buffer [50 mM Tris-HCl, pH 8; 150 mM NaCl; 1 mM EDTA; 1 mM EGTA; 1% Triton X-100; 0.5% sodium deoxycholate; 0.1% sodium dodecyl sulfate (SDS); 50 mM NaF; 5 mM Na_2_P_4_O_7_; 1 mM phenylmethylsulfonyl fluoride, and complete protease inhibitor cocktail (Roche, Rotkreuz, Switzerland)] for 20 min at 4 °C and centrifuged at 10,000 ×*g* for 15 min The supernatants were collected, and a total of 40 µg protein was subjected to SDS-polyacrylamide gel electrophoresis (SDS-PAGE), transferred onto a polyvinylidene difluoride membrane, and revealed with the appropriate antibodies: anti-GFAP (diluted 1/1000; Sigma ref. #G3893), anti-IBA1 (diluted 1/1000; Wako ref. #019-19741), anti-BDNF (diluted 1/500; Abcam ab203573), anti-p-62 (diluted 1/500; Abcam ab56416), and anti-ACTIN (diluted 1/1000; Sigma ref. #A2066). Primary antibodies were incubated overnight at 4 °C. Images were obtained with either a FujiLAS4000 using ECL or ECL-Prime (Amersham Biosciences, GR Healthcare). The results were analyzed using the Image Studio version 5.2 software (LI-COR Biosciences, Germany). Experiments were performed in at least three individuals from each genotype.

### Statistical analyses

Continuous variables were summarized using the mean (standard deviation) and median (1st, 3rd quartiles). Categorical variables were summarized using absolute and relative frequencies (%).

To assess the effectiveness of the treatment with substances (i.e., metformin and chloroquine) in worms expressing 112Q in mechanosensory neurons, we used a beta regression model adjusted for neuronal response, including genotype, chloroquine administration, and metformin intake as interacting variables. To assess the dynamics of aggregation, we performed a negative binomial regression, which is appropriate for count data. To assess the requirement of AMPK for metformin effects in mechanosensory neurons disrupted by 112Q, a beta regression was adjusted to include genotype (*aak-2* vs. wild-type) and treatment (metformin vs. vehicle) as interacting variables. To investigate the requirement of AMPK in the aggregation of 40Q, an adjusted negative binomial was performed, including treatment with metformin, treatment with chloroquine, and genotype as interacting variables.

To investigate the effectiveness of treatment in the behavioral tests in mice (tail suspension test, rotarod, and beam balance), a mixed linear regression was performed adjusting for interactions between genotype, treatment, and phase, and including mice as a random intercept.

The analysis of the concentration of phosphorylated ACC and p62 expression was performed through beta regression models, including treatment and genotype as interacting variables. To assess BDNF expression, we used a beta mixed- effect model, with mouse codes as a random intercept, treatment, and genotype included as interacting variables and tissue included as a confounding variable. To evaluate IBA1 and pERK1/2 expression, beta mixed-effect models were used, with mouse codes as a random intercept and treatment and genotype included as interacting variables. Analysis of GFAP expression was performed through a beta regression that included treatment and genotype as interacting variables and tissue as a confounding variable.

To analyze the presence of mHtt aggregates, a mixed beta regression was performed, including mouse codes as a random intercept and treatment and genotype as interacting variables. Brain region (i.e., cortex and striatum) was used as a confounding variable.

All statistical analyses were performed using R (3.4.4) and the packages clickR (0.3.64), betareg (3.1), lme4 (1.1), MASS (7.3), and glmmTMB (0.2.1). We present the estimate (linear regression models) or odds ratio (OR, beta and negative binomial models), 95% confidence interval (95% CI) and *p* values to show statistical significance. The CI indicates a range of values that is likely to encompass the true value and is expressed as the estimate value ± 1.96 times the standard deviation.

## Results

### Metformin requires AMPK and lysosomal function to prevent aggregation of polyQ in *C. elegans*

**Fig. 1:**
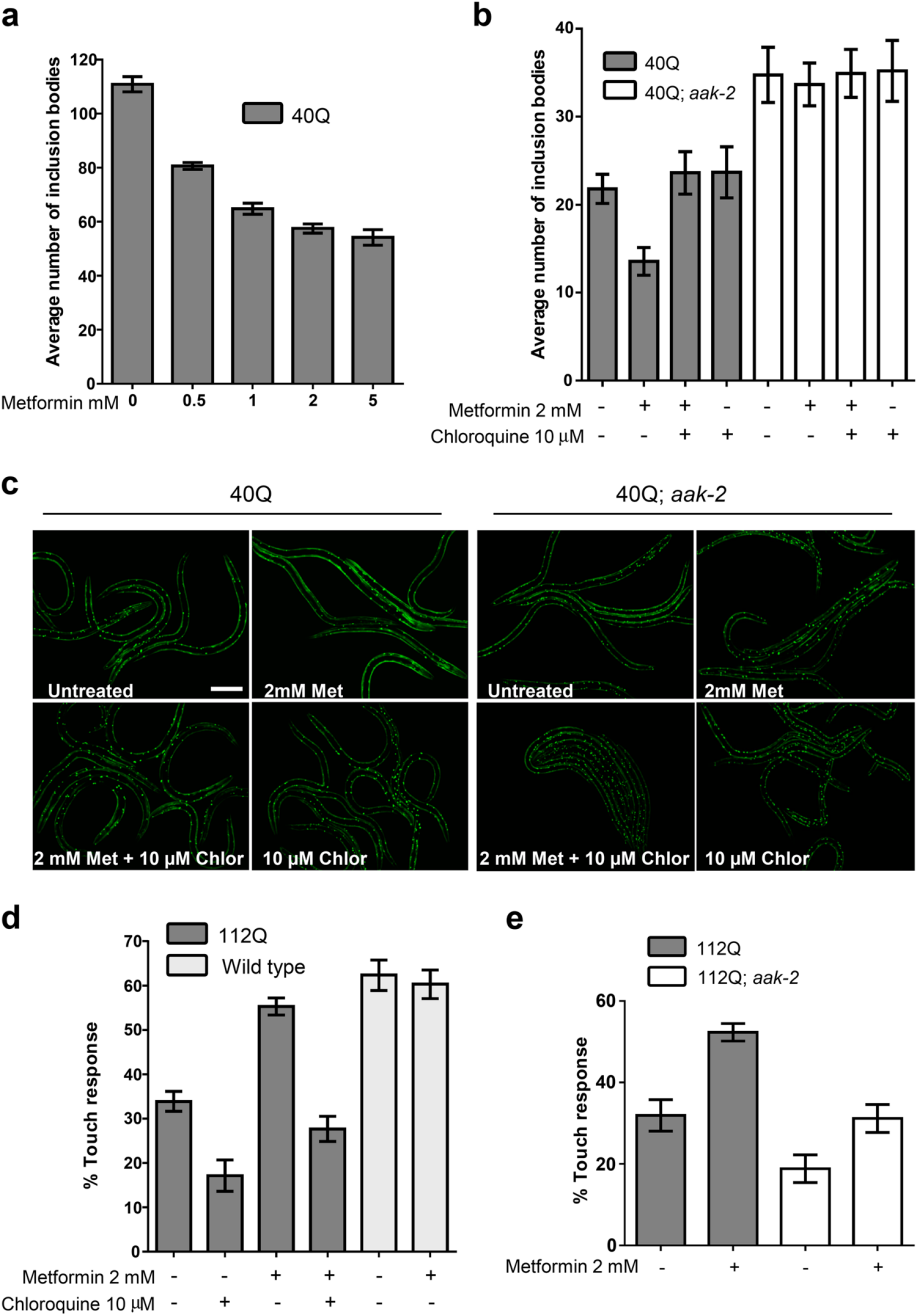
PolyQ toxicity in *C. elegans* is reduced upon AMP-activated protein kinase (AMPK) activation and requires lysosome function. **a** Metformin was able to reduce aggregation in a dose-dependent manner in 1-day-old adult worms expressing 40Q::YFP in muscle cells. Treatment with metformin significantly rescued aggregates in a dose-dependent manner (*p* < 0.001). **b** Metformin required *aak-2*/AMPKα and lysosome function to reduce the number of inclusion bodies in L4 larvae. Larvae were used to test for AMPK function because the difference between mutant and wild-type worms is reduced as the animals age (data not shown). Treatment with metformin rescued aggregates in 40Q animals (*p* < 0.001). Chloroquine maintained high levels of inclusion bodies even when the 40Q worms are treated with metformin (*p* < 0.001). Metformin required *aak-2*/AMPK to partially rescue polyQ aggregation (*p* < 0.001). **c** Photographs of wild-type and *aak-2*/AMPKα mutant L4 larvae expressing 40Q::YFP in muscle cells treated with or without 2 mM metformin and/or 10 μM chloroquine. Larvae were used to test for AMPK function because the difference between mutant and wild-type worms is reduced as the animals age (data not shown). **d** Treatment with metformin significantly rescued neuronal function (assessed by the touch response; see Materials and methods) in 112Q-TdTomato worms (*p* < 0.001). The metformin-induced rescue of neuronal function required lysosomal function: chloroquine treatment reduced neuronal recovery even when the worms were treated with metformin (*p* = 0.261). **e** Worms require AMPKα activity to rescue the neuronal function induced by metformin. Metformin was applied to worms expressing 112Q-TdTomato in mechanosensory neurons in an *aak-2* background. The touch response was assessed as above. Metformin required *aak-2*/AMPK to rescue neuronal function (*p* = 0.634). In all cases, values are the mean, and bars indicate the confidence interval (95% CI)

*Caenorhabditis elegans* worms expressing 40Q::YFP (40Q) in muscles presented an age-dependent appearance of inclusion bodies^32^. Culturing 40Q worms with metformin caused a dose-dependent reduction in the number of inclusion bodies in adult worms (OR = 0.878, 95% CI [0.862, 0.895], *p* < 0.001) (Fig. 1a). To determine whether metformin was acting through the AMPK pathway, we introduced a mutant allele of *aak-2*/AMPKα, *ok524*, in 40Q worms and observed that L4 larvae worms showed an enhanced aggregation phenotype (OR = 1.60, 95% CI [1.41, 1.81], *p* < 0.001) (Fig. 1b, c). Second, growing these worms in the presence of 2 mM metformin blocked the reduction in the number of inclusion bodies in L4 larvae (OR = 1.55, 95% CI [1.29, 1.87], *p* < 0.001) (Fig. 1b, c), suggesting that AMPK function was required for metformin to reduce aggregation of polyQs in 40Q worms.

To test whether autophagy was involved in the reduction of the number of inclusion bodies produced by metformin, the effect of the drug on 40Q-derived worms either treated or not treated with chloroquine, a drug that prevents lysosome function, was analyzed^33^. Chloroquine treatment inhibited the ability of metformin to reduce the number of inclusion bodies in L4 larvae (OR = 1.60, 95% CI [1.34, 1.92], *p* < 0.001) (Fig. 1b, c), suggesting that autophagy was required along with AMPK function to reduce the aggregation of polyQs in *C. elegans*.

### Metformin treatment ameliorates neuronal toxicity in an AMPK- and lysosome-dependent mechanism

Next, tests were performed to determine whether the metformin-induced reduction in the number of mHtt inclusions correlated with a restoration of neuronal functionality. With this aim, a new model of polyQ toxicity in mechanosensory neurons was created (Additional file 1: Fig. S1). This model consists of a transgene that expresses a tandem of 112 glutamines fused with TdTomato in mechanosensory neurons^23^. This fusion protein produces aggregates, as confirmed by the presence of inclusion bodies in mechanosensory neurons (Additional file 1: Fig. S1b), and induces neuronal toxicity, indicated by compromised mechanosensation in the tail of these transgenic animals (Additional file 1: Fig. S1c). Metformin treatment of these animals induced a clear improvement in the touch response (Fig. 1d), reaching values similar to WT performance (OR = 2.46, 95% CI [1.9, 3.19], *p* < 0.001), but the improvement in the touch response induced by metformin was reduced in the presence of chloroquine (*p* = 0.26) (Fig. 1d). In addition, when we used a *C. elegans* model that lacked the AMPK catalytic subunit (112Q *aak-2*/AMPKα mutants), the worms exhibited a reduced touch response (OR = 0.36, 95% CI [0.3, 0.5], *p* < 0.001) (Fig. 1e). More importantly, although metformin treatment showed a tendency to recover this parameter, which could be explained by alternative AMPK-independent actions of metformin, the effect was not statistically significant (*p* = 0.83) (Fig. 1e). All these results indicated that the beneficial effects of metformin required proper AMPK and lysosomal functions.

### Metformin reduces early behavioral defects in a mouse model of HD

**Fig. 2:**
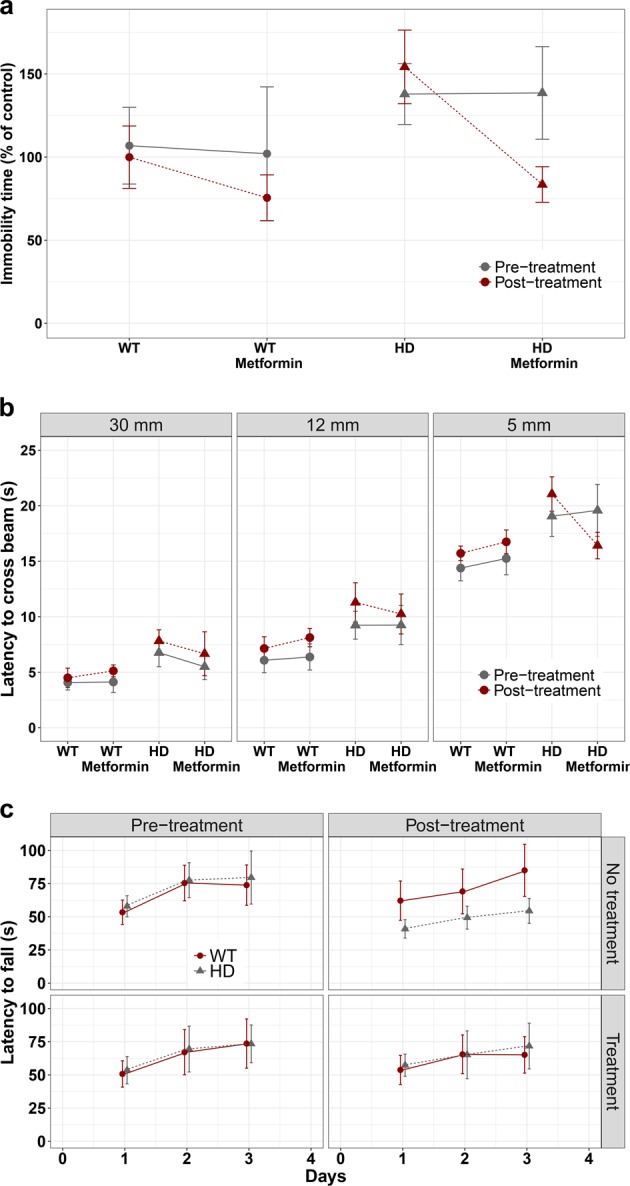
Metformin treatment ameliorates the neuropsychiatric and motor behavior phenotype in heterozygous zQ175 mice. Different behavioral tests were analyzed in 3-month-old mice (pretreatment) and after 3 months of treatment (6-month-old; post treatment). **a** Huntington disease (HD) and control mice were subjected to the tail suspension test, and the immobility time was measured (see Materials and methods). HD mice showed a depressive state as early as 3 months of age, which was worse in 6-month-old animals. However, when the mice were treated with metformin, their depression-related behavior became similar to that in wild-type (WT) animals (*p* < 0.037). **b** HD and control animals were subjected to the beam balance test. The time to cross each beam was recorded. HD mice exhibited difficulties crossing beams of different widths compared to WT animals (30 mm: *p* < 0.001; 12 mm: *p* < 0.001; and 5 mm: *p* < 0.001). In contrast, HD mice treated with metformin showed a reduced latency to cross the beam in comparison to non-treated mice (*p* = 0.014), and the values were similar to those for WT controls in the case of the 5 mm width beam (*p* = 0.822). **c** Rotarod experiments were performed with HD and control mice as described in the Materials and methods. Three-month-old HD animals had similar motor coordination to WT mice, although this motor behavior worsened with age (*p* < 0.001). However, when HD animals were treated with metformin, they showed higher latency to fall (*p* < 0.001), and they maintained a similar motor behavior to WT mice (*p* = 0.795). Values are the mean, and bars indicate the confidence interval (95% CI)

We and others have shown that AMPK is a potential druggable target for the treatment of HD^9,17,18^. Due to the neuroprotective effects of metformin in *C. elegans* models of polyQ toxicity described above, we set up a preclinical assay (Additional file 1: Fig. S2) using the zQ175 KI mouse model of HD^34^; these mice carry a humanized exon 1 of *HTT* containing an ~190 CAG tandem repeat^34^. Three-month-old heterozygous zQ175 mice and controls were treated with 2 mg/ml metformin as described in the Materials and methods, and a battery of behavioral tests was performed at the beginning and at the end of the treatment period. As some of the earliest deficits observed in HD mice are neuropsychiatric changes, the tail suspension test was implemented to assess depression-related behaviors^35–37^. This test is used to model behavioral despair by measuring immobility time during testing^38^. Analysis using mixed linear regression models of the generated data identified great evidence of depressive behavior in HD mice compared to that in WT mice (normalized to 100%) at 3 months of age (Estimate = 30.89, 95% CI [5.451, 56.33], *p* = 0.023) (Fig. 2a, comparison of gray circles, WT, with gray triangles, HD). Three months later, the symptoms were aggravated in untreated HD mice (Fig. 2a, red triangles), in contrast to HD mice treated with metformin, which demonstrated depression-related behavior similar to that in WT animals (Estimate = −51.9, 95% CI [−98.4, −5.4], *p* = 0.037) (Fig. 2a, comparison of red circles, WT, with red triangles, HD).

To further characterize the effect of metformin on early behavioral changes, we subjected HD and WT mice to two motor tests: the beam balance and rotarod tests, and mixed linear regression models were performed to analyze the data. Untreated HD mice took longer to cross the three types of beams (30, 12, and 5 mm) both at 3 and 6 months of age [(Estimate 30 mm = 2.701, 95% CI [1.3, 4.1], *p* < 0.001; Estimate 12 mm = 3.29, 95% CI [1.6, 5.0], *p* < 0.001; Estimate 5 mm = 4.68, 95% CI [2.9, 6.5], *p* < 0.001)] (Fig. 2b). However, in metformin-treated HD animals, an amelioration of the capacity to cross the 5-mm-wide beam was observed (Estimate = −5.3, CI 95% [−9.3, −1.3], *p* = 0.014) (Fig. 2b).

Analysis of the rotarod data showed that 3-month-old HD animals did not present any motor impairment compared to their WT littermates (*p* = 0.24) (Fig. 2c). However, at 6 months of age, untreated HD animals performed worse than the WT groups (Estimate = −25.4, 95% CI [−47.5, −6.4], *p* < 0.001) (Fig. 2c), suggesting a motor impairment that progressed over time. Again, treatment of the HD mice with metformin reduced motor symptoms to levels similar to those in the WT mice (Estimate = 27, 95% CI [4.3, 55], *p* < 0.001) (Fig. 2c), demonstrating that metformin slowed the progression of the motor phenotype.

### Metformin crosses the blood–brain barrier and induces molecular changes in the brains of treated mice

After behavioral analysis, HD and control mice were euthanized, and brain samples were collected for immunostaining analyses to investigate the effect of metformin in these animals. This study was carried out through analysis of the expression of a battery of well-known markers of HD progression. We chose immunostaining to detect biomarkers because some of these molecules tend to accumulate in specific neurons and, sometimes, in specific subcellular locations. Only immunostaining allows the detection of these markers with specific antibodies, as detection of these markers would be very difficult if the protein of interest is diluted in a preparation from the whole tissue.

**Fig. 3:**
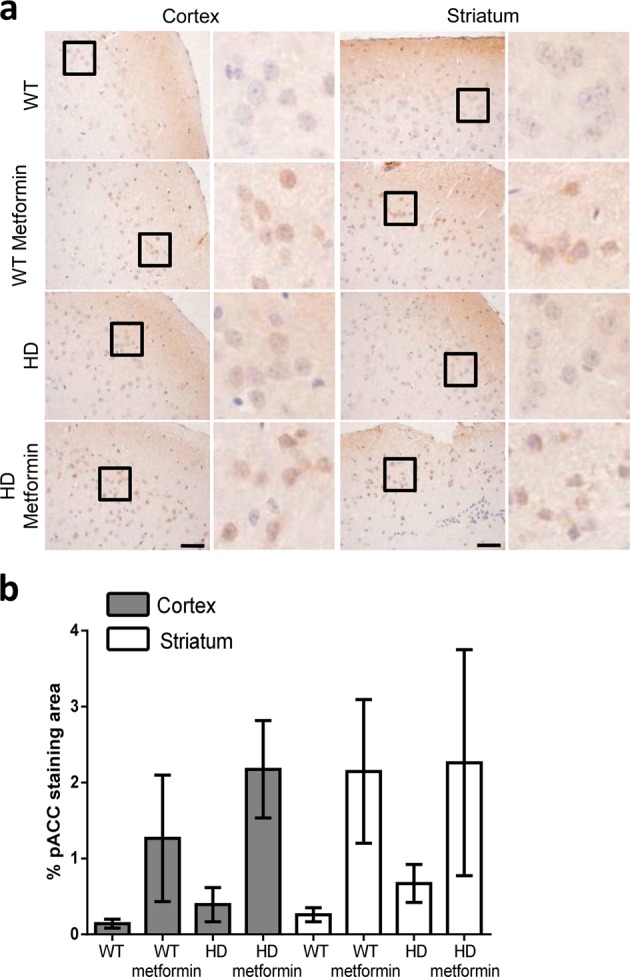
Levels of phospho-acetyl CoA carboxylase (pACC), a substrate of AMP-activated protein kinase (AMPK), in brain tissue of 6-month-old control and zQ175 mice treated or not with metformin. **a** Immunohistochemical (IHC) staining illustrates the expression levels of pACC in the cortex and striatum of zQ175 (Huntington disease (HD)) mice and their corresponding controls (wild type (WT)). Representative images of each condition are shown (bars: 50 µm). Boxed areas are enlarged, with a higher magnification, on the right side of each image. **b** The bottom panel shows a semiquantitative analysis of IHC staining using the ImageJ software. Samples from four independent mice from each group were analyzed, which generated 7 cortex and 12 striatum pictures from untreated wild-type (WT) mice, 9 cortex and 12 striatum pictures from treated wild-type (WT metformin) mice, 7 cortex and 6 striatum pictures from untreated zQ175 (HD) mice, and 10 cortex and 6 striatum pictures from HD-treated (HD metformin) mice. The analyzed area in each picture was 90,488 μm^2^. Treated mice showed increased levels of pACC, independent of their genotype (*p* < 0.001). Values are the mean, and bars indicate the confidence interval (95% CI)

First, we investigated whether metformin was able to reach the brains of mice. To do so, we chose to evaluate the levels of pACC, a typical AMPK substrate. Analysis of this species showed that cortex and striatum sections from mouse brains treated with metformin contained higher levels of pACC than those sections from untreated mouse brains (OR = 5, 95% CI [3, 8.6], *p* < 0.001) and that this difference was independent of the genotype (*p* = 0.344) (Fig. 3). These results were consistent with previous reports that indicated that metformin was able to cross the blood–brain barrier^17,39^ and confirmed that the concentration of metformin used in the assay was sufficient to induce molecular changes.

### Metformin reduces mHtt aggregation in the brains of HD mice

**Fig. 4:**
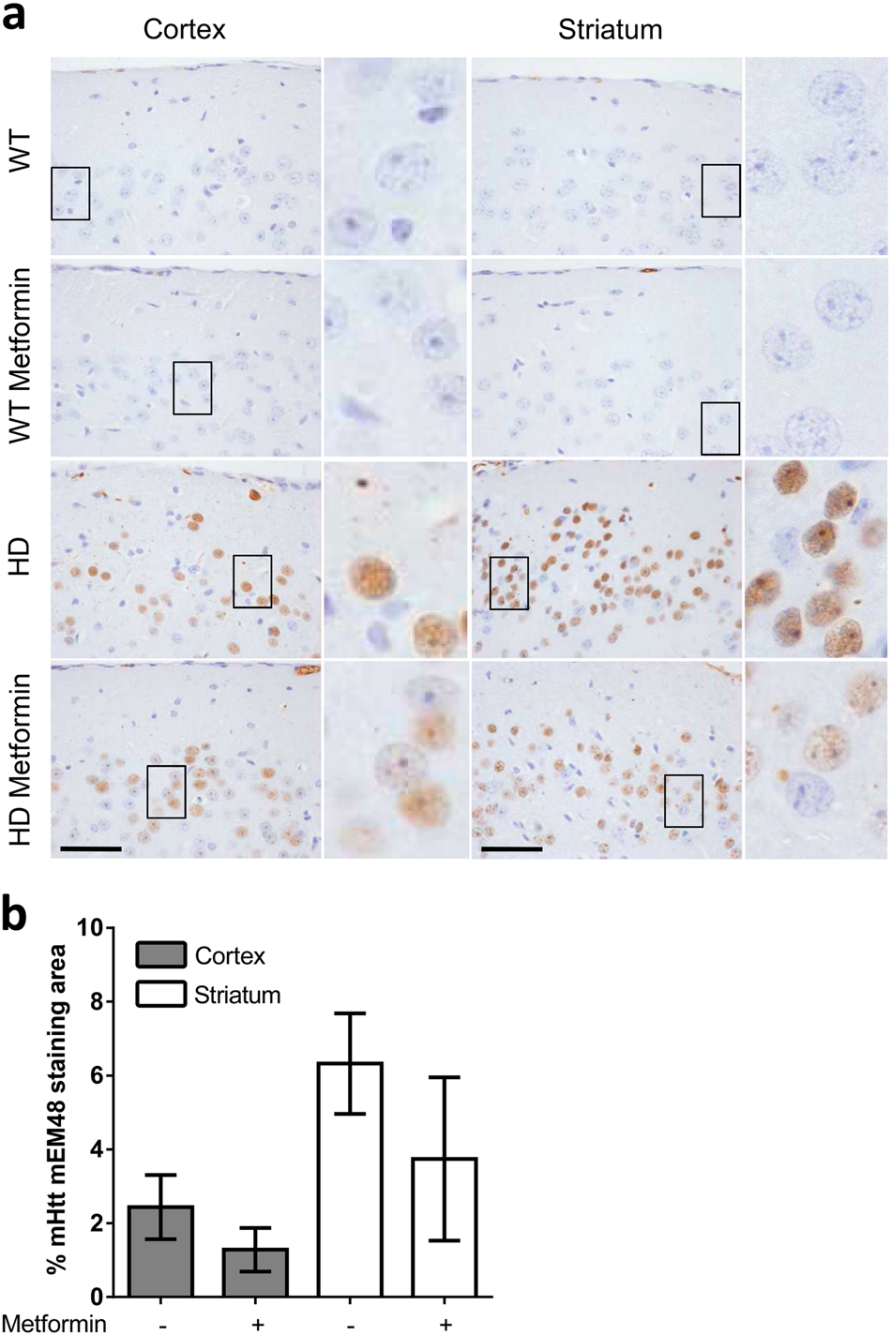
Mutant huntingtin aggregation in the brain tissue of six-month-old zQ175 mice is reduced by metformin treatment. **a** Immunohistochemical (IHC) staining illustrates the expression levels of mutant huntingtin (mHTT) in the cortex and striatum of zQ175 mice and their corresponding controls (wild type (WT)). Representative images of each condition are shown (bars: 50 µm). Boxed areas are enlarged, with a higher magnification, on the right side of each image. **b** The bottom panel shows a semiquantitative analysis of IHC staining using the ImageJ software. Samples from four independent mice from each group were analyzed, which generated 10 cortex and 8 striatum pictures from untreated zQ175 (Huntington disease (HD)) mice and 10 cortex and 7 striatum pictures from HD-treated (HD metformin) mice. The analyzed area in each picture was 90,488 μm^2^. HD mice treated with metformin showed fewer mHtt aggregates (*p* = 0.00243), and the reduction in the aggregates was more pronounced in the striatum of these mice (*p* < 0.001). Values are the mean, and bars indicate the confidence interval (95% CI)

**Fig. 5:**
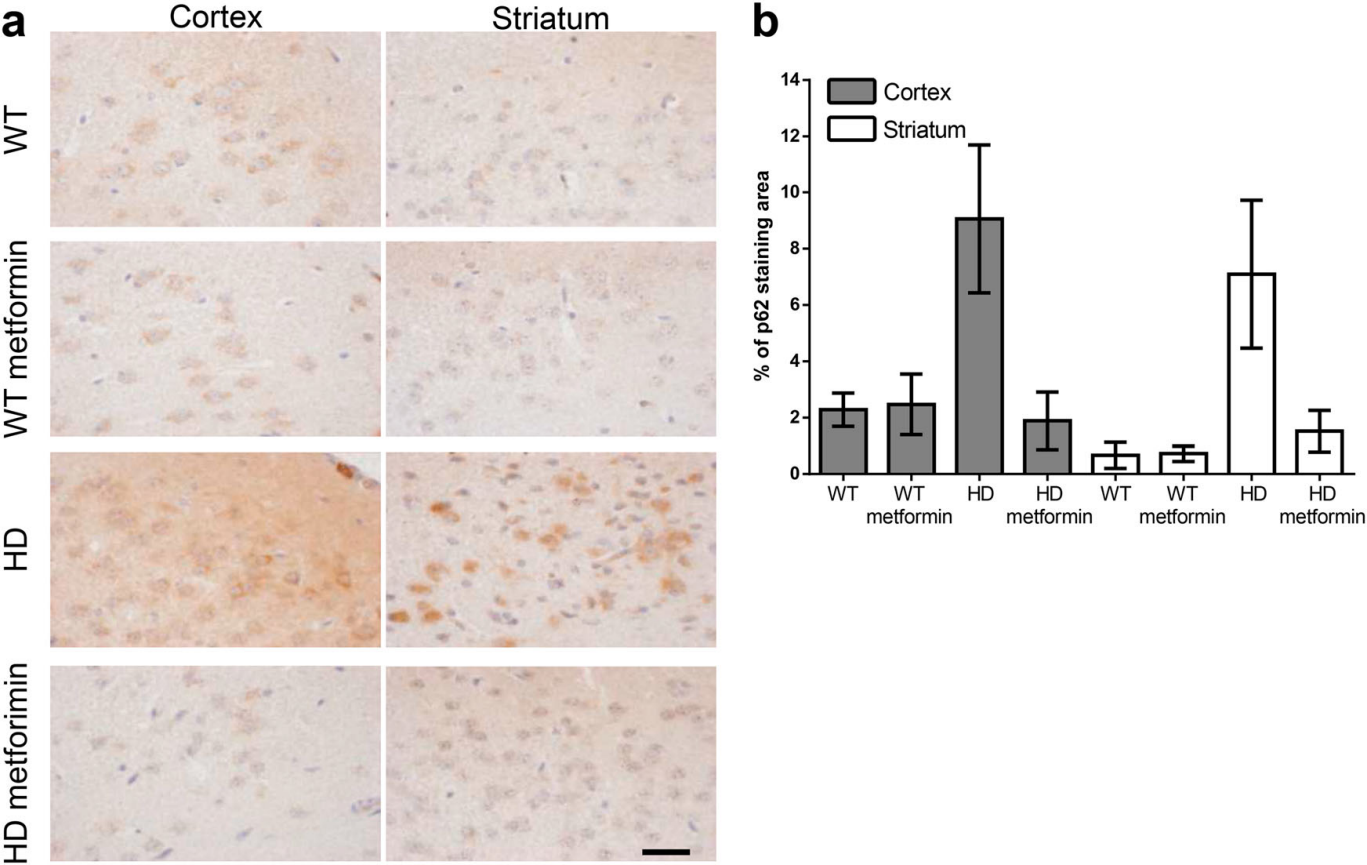
Levels of autophagy receptor p62 in brain tissue of 6-month-old control and zQ175 mice treated or not with metformin. **a** Immunohistochemical (IHC) staining illustrates the expression levels of p62 in the cortex and striatum of zQ175 (Huntington disease (HD)) mice and their corresponding controls (wild type (WT)). Representative images of each condition are shown (bars: 50 µm). **b** Semiquantitative analysis of IHC staining using the ImageJ software. Samples from four independent mice from the WT and nontreated HD groups and 5 mice from the treated HD group were analyzed, which generated 13 cortex and 8 striatum pictures from untreated wild-type (WT), 12 cortex and 7 striatum pictures from treated wild-type (WT metformin), 14 cortex and 7 striatum pictures from untreated zQ175 (HD), and 14 cortex and 13 striatum pictures from HD-treated (HD metformin) mice. The analyzed area in each picture was 90,488 μm^2^. HD mice showed higher levels of p62 than WT mice (*p* < 0.001), and treatment with metformin reduced the levels of p62 (*p* < 0.001) to values similar to those in WT mice. Values are the mean, and bars indicate the confidence interval (95% CI)

As zQ175 mice showed mHtt aggregates in the brain, we investigated the effect of metformin on these species. We used mEM48, an antibody against Htt, to explore the presence of insoluble mHtt aggregates and soluble mHtt in the striatal nucleus and cortex, which are the regions especially affected in patients and murine models of HD^40^. The Immunohistochemical (IHC) results illustrated that in these key areas, there was a substantial accumulation of nuclear mHtt aggregates that was not present in the controls (Fig. 4). However, in metformin-treated HD animals, the accumulation of aggregates was reduced (OR = 0.53, 95% CI [0.36, 0.80], *p* = 0.024) (Fig. 4), suggesting that metformin may activate some mechanism of aggregate removal, possibly autophagy. Furthermore, we found that the levels of p62 (also assessed by IHC), an autophagy receptor protein that accumulates upon autophagy impairment, were higher in the cortex and striatum of untreated HD animals than in those of controls (OR = 5.4, 95% CI [3.3, 8.8], *p* < 0.001), but metformin was able to prevent this accumulation (OR = 0.17, 95% CI [0.1, 0.3], *p* < 0.001]) (Fig. 5), suggesting a recovery of autophagy function.

### Metformin prevents the loss of BDNF levels present in HD mice

**Fig. 6:**
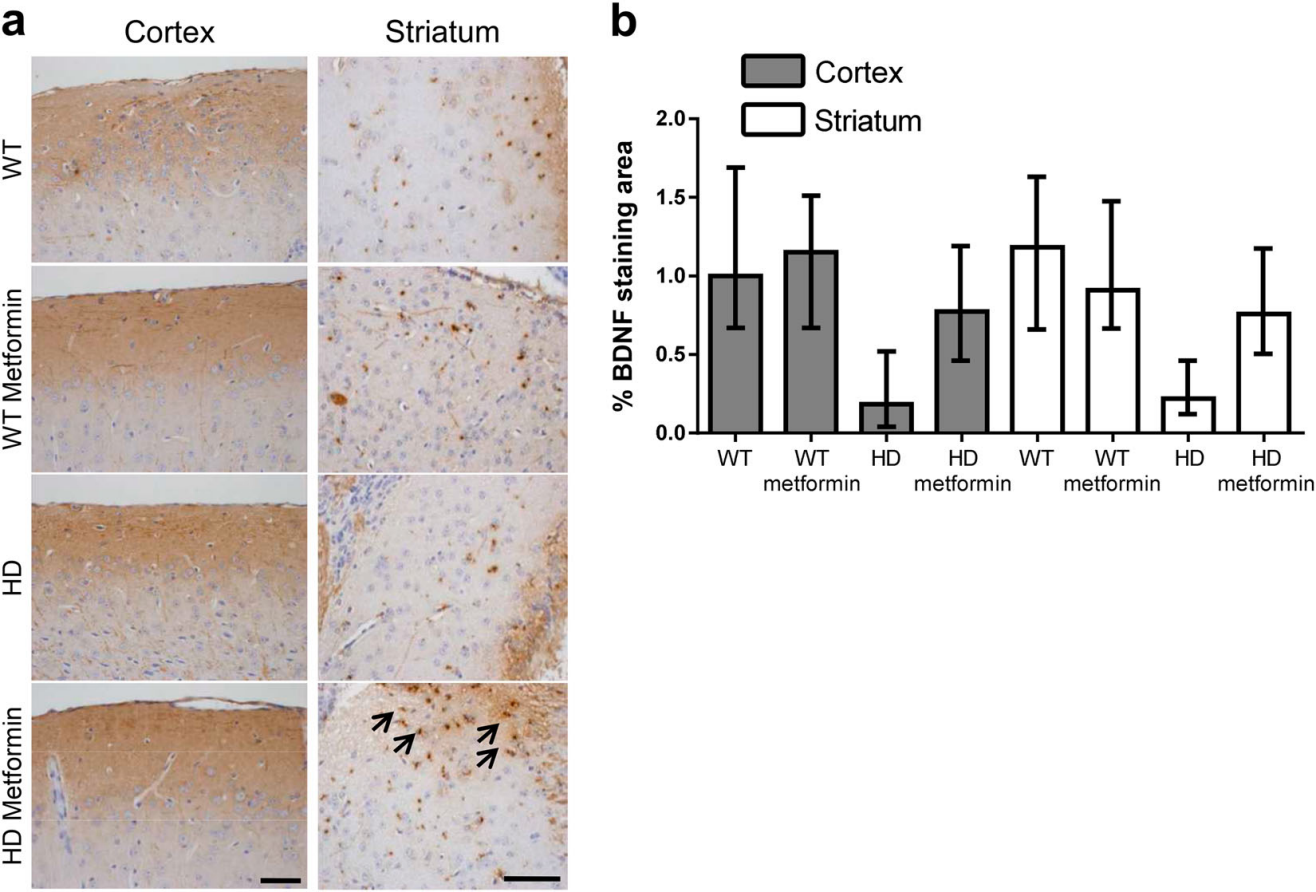
Metformin prevents the loss of brain-derived neurotrophic factor (BDNF) levels in Huntington disease (HD) mice. **a** Immunohistochemical (IHC) staining of the striatum of HD and control mice using anti-BDNF antibodies. Arrows denote the localization of BDNF in treated HD samples. Representative images of each condition are shown (bar: 50 µm). **b** Semiquantitative analysis of IHC staining using the ImageJ software. Samples from four independent mice from wild-type (WT) and nontreated HD groups and five mice from the treated HD group were analyzed, which generated 4 cortex and 4 striatum pictures from untreated wild-type (WT), 4 cortex and 4 striatum pictures from treated wild-type (WT metformin), 4 cortex and 4 striatum pictures from untreated zQ175 (HD), and 5 cortex and 5 striatum pictures from HD-treated (HD metformin) mice. The analyzed area in each picture was 90,488 μm^2^. The protein levels of BDNF in HD mice were lower than those in WT littermates (*p* < 0.001), and metformin treatment increased the protein levels (*p* < 0.001). HD mice treated with metformin showed similar levels of BDNF as WT littermates (*p* = 0.853). Values are the mean, and bars indicate the confidence interval (95% CI)

Striatal neurons in the brain require BDNF for their activity and survival, and this factor protects neurons of the striatum from mHtt-induced toxicity^41^. Interestingly, this neurotrophic factor is reduced in patients^42–44^ and models of HD, including zQ175 mice^45^. This observation led to the hypothesis that impaired production of BDNF may contribute to degeneration of neurons in the striatum^46^. In agreement with previous results^45^, IHC staining of the cortex and striatum of 6-month-old animals illustrated a remarkable decrease in BDNF expression in HD mice (OR = 0.21, 95% CI [0.01, 0.39], *p* < 0.001) (Fig. 6). Interestingly, treatment with metformin ameliorated the levels of BDNF in the cortex and striatum of HD mice (OR = 3.63, 95% CI [1.76, 7.49], *p* < 0.001) (Fig. 6). This increase in BDNF expression may account, at least in part, for the restored neuronal function shown by the behavioral assays of metformin-treated HD mice described above.

### Brains from zQ175 mice show signs of inflammation that can be prevented by treatment with metformin

Although HD‐related research is primarily focused on neurons, glial cells are also important because of their critical role in brain function. Astrocytes and microglia directly regulate synaptic communication and the function of the blood–brain barrier^47^ and are the main actors in the inflammatory response, alterations of which are another hallmark of HD^48^. In this regard, zQ175 animals have been shown to exhibit signs of inflammation in the nervous system at early stages of disease development^49^.

**Fig. 7:**
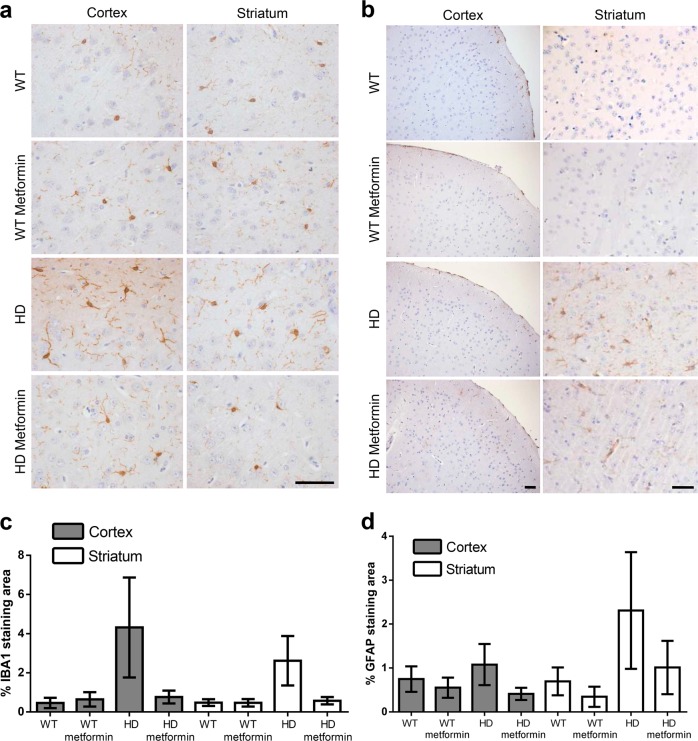
The inflammatory response in the brain tissue of 6-month-old zQ175 mice is reduced after metformin treatment. Immunohistochemical (IHC) staining of the striatum of Huntington disease (HD) and control mice using anti-ionized calcium-binding adaptor molecule 1 (IBA1) (**a**) and anti-glial fibrillary acidic protein (GFAP) (**b**) antibodies. IHC staining of IBA1 and GFAP were significantly reduced by metformin treatment. Representative images of each condition are shown (bar: 50 µm). **c**, **d** The semiquantitative analysis of IHC staining of both biomarkers using the ImageJ software. The number of independent mice and samples from each is as follows: (1) for GFAP, we analyzed 4 nontreated wild type (WT), 3 treated WT, 4 nontreated HD, and 5 treated HD mice, which generated 10 cortex and 8 striatum pictures from untreated wild-type (WT), 6 cortex and 8 striatum pictures from treated wild-type (WT metformin), 4 cortex and 6 striatum pictures from untreated zQ175 (HD), and 7 cortex and 10 striatum pictures from HD treated (HD metformin) mice. The analyzed area in each picture was 361,920 μm^2^; (2) for IBA1, we analyzed 4 mice from each group (nontreated and treated WT mice and nontreated and treated HD mice), which generated 4 cortex and 10 striatum pictures from untreated wild-type (WT), 7 cortex and 5 striatum pictures from treated wild-type (WT metformin), 6 cortex and 9 striatum pictures from untreated zQ175 (HD) and 11 cortex and 13 striatum pictures from HD treated (HD metformin) mice. The analyzed area in each picture was 36,580 μm^2^. **c** Analysis of these data indicates that HD mice expressed higher levels of IBA1 than WT mice in both the cortex and striatum (*p* < 0.001 in both cases) and that metformin reduced the levels of IBA1 to those of WT mice (*p* = 0.932). **d** HD mice expressed higher levels of GFAP than WT mice (*p* < 0.001). Treatment with metformin significantly reduced GFAP levels in HD mice to levels similar to those of WT mice (*p* = 0.33). Values are the mean, and bars indicate the confidence interval (95% CI)

Microglial activation in tissue specimens is typically characterized by increased numbers of cells and morphological changes from their normal scanning mode into an immune-activated phenotype. These changes in reactive microglia were investigated using an antibody against IBA1, which is expressed specifically in these cells^50^. Thus, increases in IBA1 staining most likely represent activated or proliferating microglia^51^. Figure 7a illustrates IHC staining analyses of the expression levels of IBA1 in the striatum and cortex of control and HD mice at 6 months of age. In the untreated HD mice, microglia were more numerous than in the control mice (OR = 5.2, 95% CI [3.25, 8.33], *p* < 0.001) (Fig. 7c). In contrast, these features were significantly reduced in HD mice treated with metformin (OR = 0.22, 95% CI [0.12, 0.43], *p* < 0.001) (Fig. 7c). In parallel to IBA1 staining, we used an antibody targeting GFAP, an intermediate filament that is a marker of reactive astrocytes (Fig. 7b). We observed an increase in the number of reactive astrocytes in the cortex and striatum of untreated HD mice in comparison to those of controls (OR = 2.25, 95% CI [1.53, 3.3], *p* < 0.001), and metformin treatment reduced the number of reactive astrocytes (OR = 0.57, 95% CI [0.36, 0.925], *p* = 0.022) (Fig. 7d). These results indicated that metformin was able to reduce the presence of reactive astrocytes and microglia and thus reduce inflammation in these areas of the brain.

### Brain areas of HD mice show upregulation of pERK that can be prevented by treatment with metformin

**Fig. 8:**
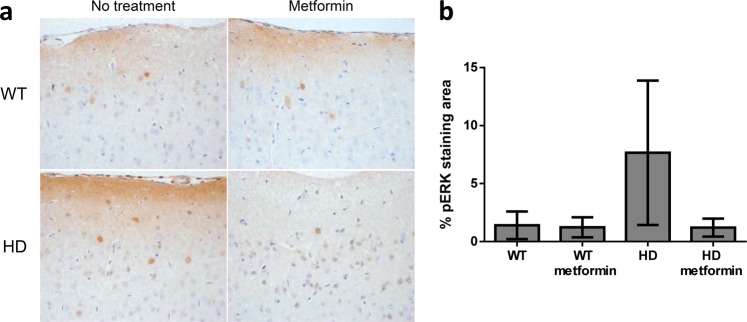
Metformin treatment reduces phospho-extracellular signal-regulated kinase1/2 (pERK1/2) expression in Huntington disease (HD) mice. **a** Immunohistochemical (IHC) staining of the cortex of Huntington disease (HD) and control mice using the anti-pERK1/2 antibody. Representative images of each condition are shown (bar: 50 µm). **b** Semiquantitative analysis of IHC staining using the ImageJ software. Samples from four independent mice from each group, except for HD-treated mice, of which we used six mice, were analyzed. These mice generated the following material: 12 cortex pictures from untreated wild-type (WT), 12 cortex pictures from treated wild-type (WT metformin), 12 cortex pictures from untreated zQ175 (HD), and 18 cortex pictures from HD treated (HD metformin) mice. The analyzed area in each picture was 90,488 μm^2^. HD mice showed higher levels of pERK1/2 than WT mice (*p* = 0.011). Treatment with metformin reduced pERK1/2 levels (*p* = 0.013) to levels similar to those in WT mice (*p* = 0.686). Values are the mean, and bars indicate the confidence interval (95% CI)

Extracellular signal-regulated MAP kinase (ERK) is at the crossroad of several signaling pathways. ERK is activated by phosphorylation when the cells are subjected to different stress conditions and triggers different signaling pathways that allow the cells to adjust to the environmental conditions. In agreement with previous results^52^, we found higher levels of pERK1/2 in the cortex of HD mice than in that of control mice (OR = 2.53, 95% CI [1.24, 5.19], *p* = 0.011) (Fig. 8), indicating that the brain of these mice was subjected to stress conditions (i.e., mHtt accumulation, inflammation, etc.). Interestingly, treatment with metformin resulted in a decrease in the levels of pERK1/2 (OR = 0.28, 95% CI [0.10, 0.76], *p* = 0.013) (Fig. 8), suggesting that alleviation of stress conditions (toxicity induced by mHtt, inflammation) may result in downregulation of the ERK pathway.

In addition to immunohistochemistry analysis of the different biomarkers, we performed Western blot (WB) experiments to assess whether there were differences in the concentrations of p62, BDNF, IBA1, and GFAP, but no significant differences among groups were found (Supplementary Fig. S6).

## Discussion

Metformin is the most commonly used drug in the treatment of type 2 diabetes^53^. Due to its massive use worldwide, observational studies have shown that patients chronically taking metformin have unexpected beneficial side effects on aging-related diseases, such as cancer^15^, nephrotic conditions^16^, and neurodegenerative diseases (see for a review, Markowicz-Piasecka et al.^54^). Additionally, activation of AMPK by metformin has been shown to alleviate neuronal impairment in worms expressing polyQs and reduces cell death in precursors of striatal cells stressed by mHtt^9^. Furthermore, patients with HD comorbid with type 2 diabetes who are taking metformin have been suggested to have better cognitive function than regular HD patients^18^, strongly indicating that metformin may be a potential drug for HD treatment. Following this rationale, a preclinical assay was performed using the zQ175 mouse model of HD. Notably, heterozygous zQ175 mice, in contrast to other models of HD, show robust reproducible neurodegenerative phenotypes^34,55^: zQ175 animals show progressive decline in motor function^49^, cognitive impairment^34,49,56^, and neuropsychiatric phenotypes^56^, which are the result of neuronal loss in the striatum. The data presented in this work on untreated zQ175 mice are in agreement with the motor and neuropsychiatric phenotypes described previously in this model. However, in this work, we present strong evidence that the administration of metformin in the early phases of HD (i.e., when animals are 3 months old) alleviates neuropsychiatric and motor phenotypes in zQ175 mice, suggesting that metformin may reduce neuronal toxicity caused by mHtt aggregation in the striatum and cortex of these animals. As the number of CAG repeats in treated and untreated animals was similar (Supplementary Figs. S4, S5 and Table S1), we assumed that the obtained results were due to the effect of metformin and not due to differences in the number of CAG repeats present in each animal.

We investigated several biomarkers to determine the mechanism by which metformin induces functional recovery of behavior in HD mice. First, we determined that metformin was able to reach the brains of treated mice, as judged by the increase in the expression of pACC, a typical AMPK substrate. These results were consistent with previous reports that indicated that metformin was able to cross the blood–brain barrier^17,39^ and activate AMPK in the brain. Second, staining of mHtt showed that 6-month-old HD mice had substantial nuclear aggregation, in agreement with previous work^57^. In contrast, metformin treatment reduced the number of aggregates, suggesting that this drug was capable of enhancing a protein clearance pathway. These results were in agreement with those previously reported by Vázquez-Manrique et al.^9^ showing that activating AMPK by genetic means induced a reduction in mHtt in an in vitro model of HD. The reduction in the levels of mHtt produced by metformin could be due to an enhancement of autophagy, probably mediated by AMPK activation. Furthermore, we show evidence of a decrease in the levels of the autophagy receptor protein p62 in HD mice upon metformin treatment. As this protein accumulates under conditions of reduced autophagy, our results suggest that metformin enhances the autophagy pathway. While this work was under revision, a study was published indicating an alternative mechanism of action of metformin^58^. According to these authors, metformin reduces the translation rate of mHtt via inhibition of a pathway involving mammalian target of rapamycin (mTOR). With this possibility in mind, we suggest that metformin could decrease mHtt levels either by decreasing its synthesis by an mTOR-dependent pathway or by increasing autophagy through an AMPK-dependent pathway or both. We would like to note that these two mechanisms are not independent because activation of AMPK is well known to also lead to inhibition of the mTOR pathway^59^.

To gain further insight into the mechanism of action of metformin, two *C. elegans* models of polyQ toxicity were used to investigate whether AMPK and autophagy could be involved in the beneficial effects of metformin. Through this examination, this drug was found to reduce the aggregation of polyQs in muscle cells and restore function to mechanosensory neurons stressed by polyQs, with these effects dependent on AMPK function. In addition, the beneficial effects of metformin were strongly impaired when lysosome function was inhibited using chloroquine, suggesting that autophagy was necessary for polyQ clearance. These results suggest a potential mechanism of neuroprotection by metformin, in which AMPK activation and lysosomal function play a role.

In addition to mHtt clearance, we report in this work a positive effect of metformin on BDNF levels. Increased BDNF levels are strongly linked to neuronal survival^49^, and increasing the levels of this neurotrophic factor in models of HD has shown neuroprotective effects^60^. The results of this study indicate that treatment with metformin partially prevents the reduction in the levels of BDNF characteristic of HD mice, suggesting another possible mechanism of neuroprotection triggered by metformin. In contrast to immunohistochemical analysis of BDNF, WB analysis of this biomarker did not show any significant difference between treated and control mice (Supplementary Fig. S6). A possible reason for the absence of differences between the samples could be the dilution of the abnormal expression of one particular component in one specific area by the rest of the tissue in the whole extracts of the brain regions investigated. The same explanation may apply to the WB analysis of p62, IBA1, and GFAP.

An additional effect of metformin is its prevention of inflammation in the brain. Inflammation is normally an adaptive biological response to pathogen infection and tissue injury that serves to engage the immune system and tissue repair mechanisms. In response to infection or neuronal tissue damage, microglia rapidly alter their morphology and increase phagocytic activity, initiating an innate immune response by secreting various inflammatory molecules, including interleukin (IL-6) and tumor necrosis factor-α (TNFα). When the inciting insult cannot be eradicated, persistent expression of mediators, such as IL-6, IL-8, and TNFα, drive chronic inflammation and can contribute to tissue damage and disease progression (reviewed in Crotti and Glass^48^). In HD patients, astrocytes and microglia become activated, as shown by the upregulation of GFAP and IBA1, respectively, and the degree of activation correlates with disease^47^. Microglial activation is known to occur before the first symptoms of the disease become apparent, and increased numbers of GFAP‐positive astrocytes are observed as early as the first pathological stages of HD in patients^47^. In our study, we observed the same inflammatory response in untreated zQ175 mice (see Fig. 7). The presence of inflammation in HD raises the still unresolved question of whether this process is the response of surrounding cells to a neuron-autonomous degenerative process and/or due to glia-autonomous immune activation resulting from the expression of mutant Htt. In any case, we present evidence that metformin is also able to reduce the activation of astrocytes and microglia, which could reduce the inflammatory response in these HD-treated mice. In addition, we also report a decrease in the levels of pERK1/2 (a biomarker of cellular stress) upon metformin treatment, which is consistent with the neuroprotective effects of the drug.

In this work, we present strong evidence indicating that metformin alleviates neuropsychiatric and motor phenotypes in zQ175 mice, suggesting that metformin may reduce neuronal toxicity in the striatum and cortex of these animals. We also present evidence of a reduction in the levels of mHtt and inflammation and an increase in the levels of BDNF upon metformin treatment, which could be the cause of the neuroprotective properties of this drug. Because metformin has good safety records and is already approved for clinical use, we think that the translation of our results to clinical practice could be straightforward.

## Supplementary information

Supplementary Materials

## Data Availability

The datasets generated and/or analyzed during the current study are available from the corresponding author on reasonable request.
