# Supplementary material for: Metformin treatment reduces motor and neuropsychiatric phenotypes in the zQ175 mouse model of Huntington disease
Source: Exp Mol Med. 2019 Jun 5;51(6):65. doi: 10.1038/s12276-019-0264-9 (PMC6549163; doi:10.1038/s12276-019-0264-9)
Supplement: Supplementary file 1 — Supplementary Materials [file 12276_2019_264_MOESM1_ESM.pdf]

SUPPORTING INFORMATION

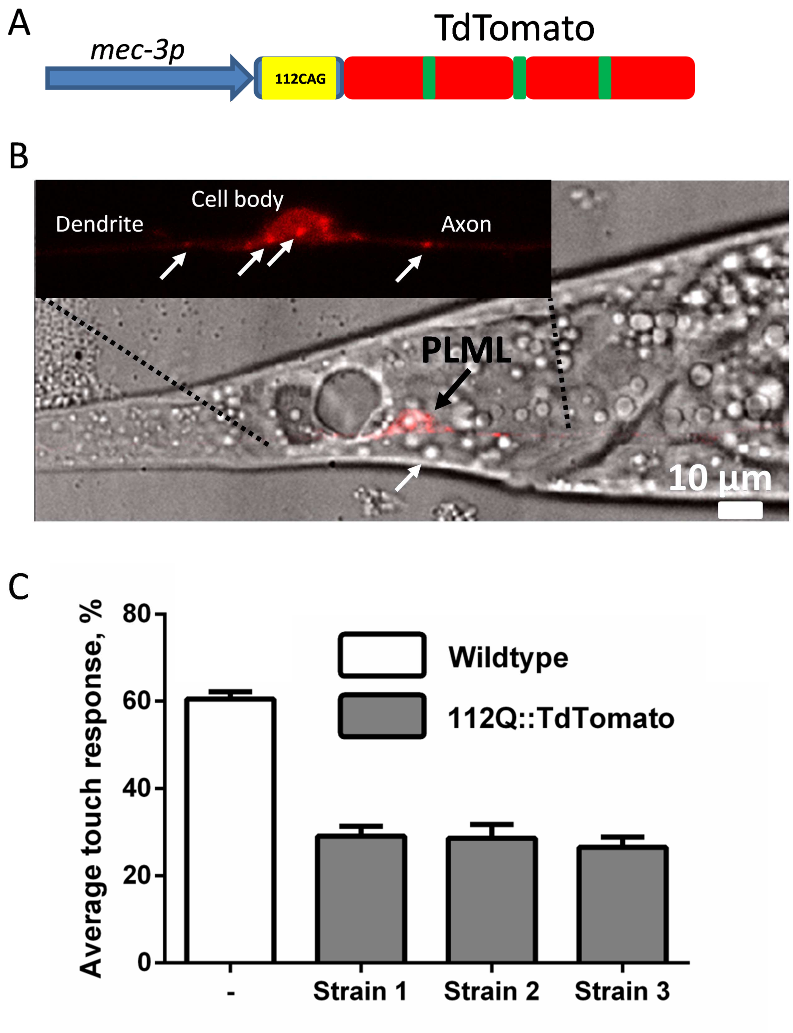

Fig. S1 *C. elegans* model of polyQ toxicity in mechanosensory neurons. A) Diagram of the construct produced to make the 112Q::TdTomato transgene. The blue arrow represents the promoter of the *mec-3* gene, which is expressed exclusively in mechanosensory neurons. The yellow bar represents the 112 CAG triplets, and is flanked by a few nucleotides of the first exon of *HTT.* Red bars represent the coding region of TdTomato, which is interrupted by three artificial introns (green bars). B) Confocal images of the tail of a worm expressing 112Q::TdTomato. The inset shows the fluorescence produced by the protein. White arrows point towards aggregates of 112Q::TdTomato. C) Touch assays in three independent transgenic strains carrying 112Q::TdTomato show reduced touch response, compared to wild type animals.

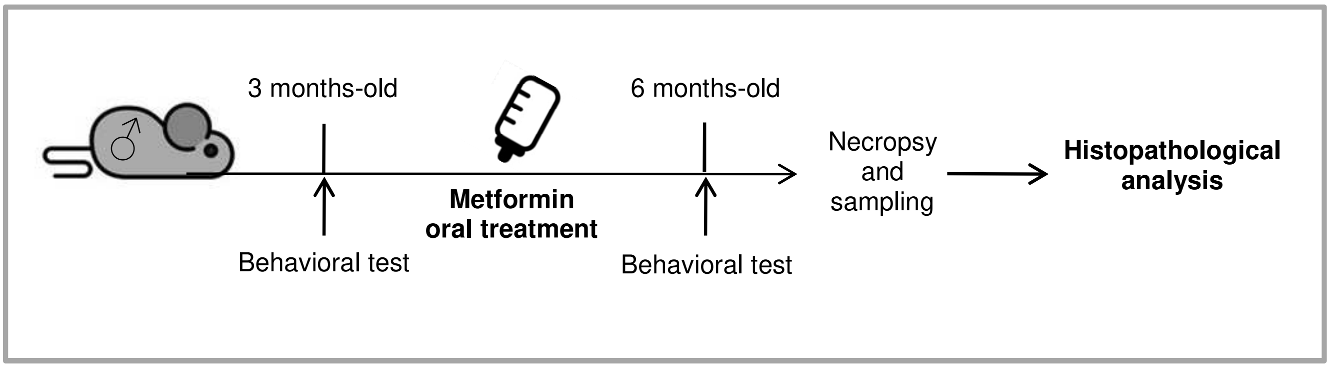

Fig. S2 Schematic diagram explaining *in vivo* working plan for testing metformin in zQ175 mice. Different behavioral test were analyzed in 3 months old mice (pre-treatment) and after 3 months of treatment (six month-old; post-treatment).

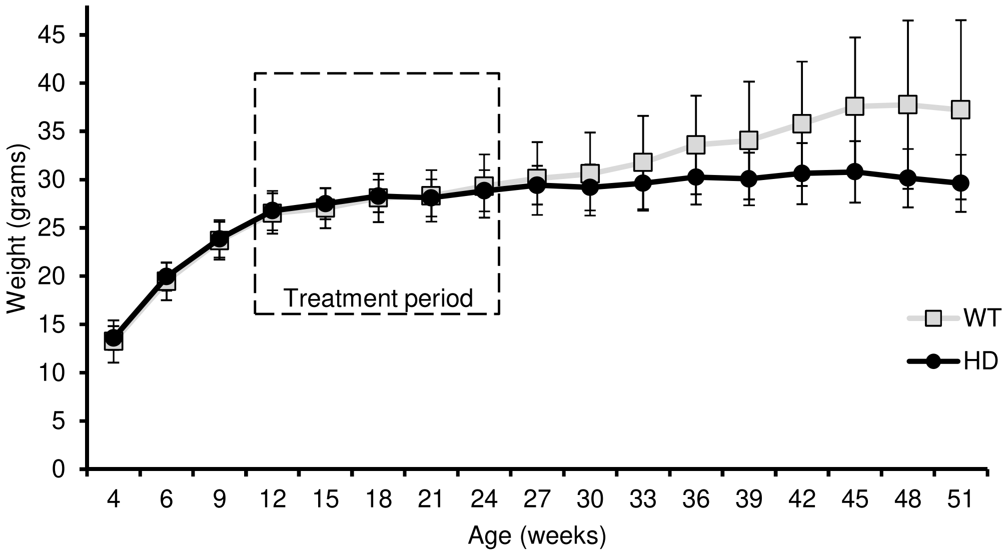

Fig. S3 Body weight of wild type and heterozygous zQ175 mice as a function of age. Body weight in WT males progressively increased with age. Body weight of HD mice remains constant during the evaluation period, being only smaller than the WT from week 42.

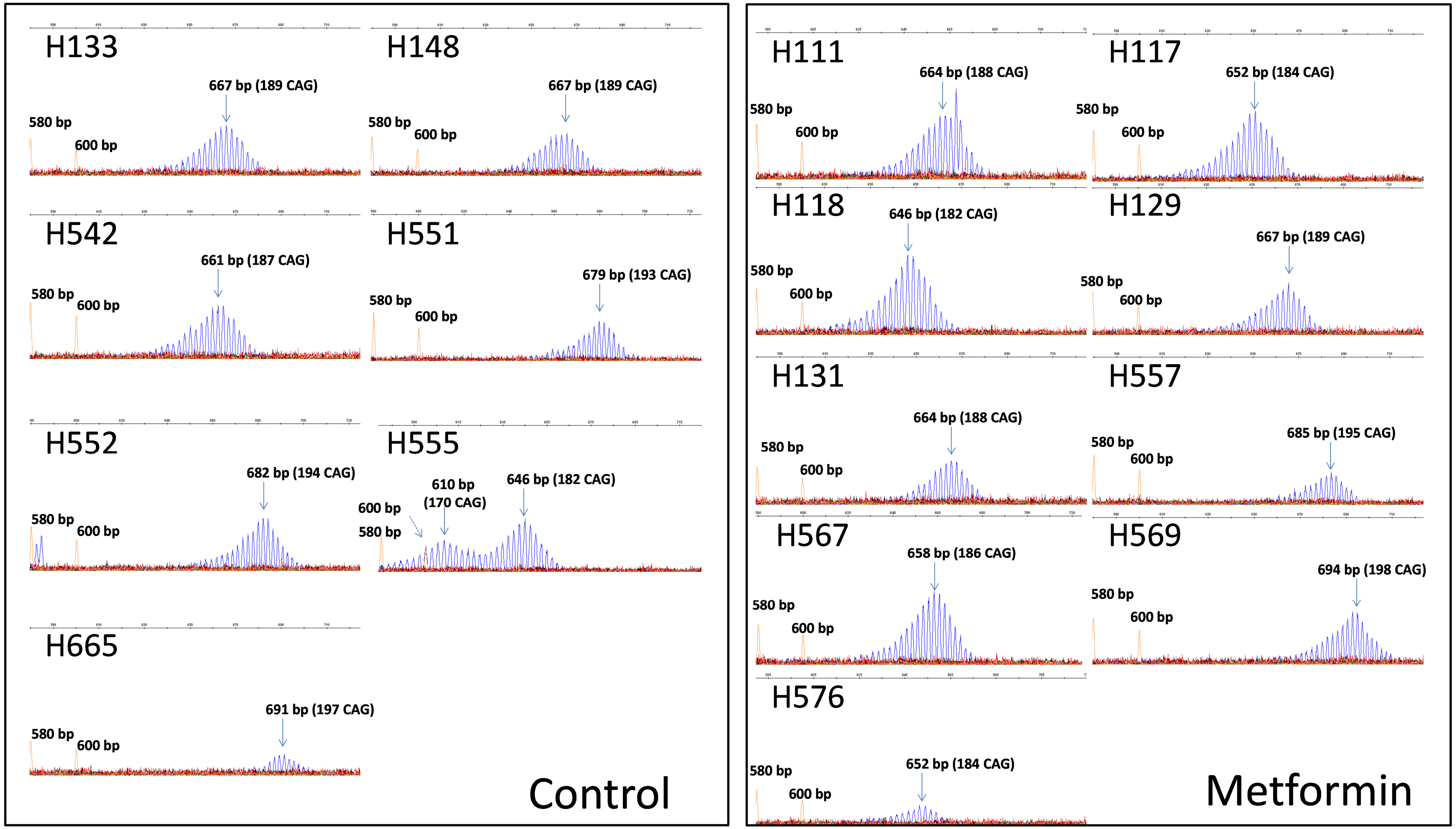

Fig. S4. Analysis of CAG expansions from genomic DNA of tail tissue from zQ175 mice. GeneMapper raw data shows that the CAG expansions from both control (n: 7) and metformin-treated (n: 9) P7-P10 old mice do not show substantial difference in length. The two peaks of 580 and 600 bp correspond to the last two bands of the GeneScan 600 LIZ dye Size Standard v2.0 (Thermofisher). We point out the most likely average expansion of CAG triplets (blue arrow) and the corresponding number of CAG repeats. Mouse H555 shows two different distributions. In mouse H111 there is one peak with higher intensity, at the right of the arrow, which could be the most representative expansion, thought it seems more likely to be and artifact.

Control

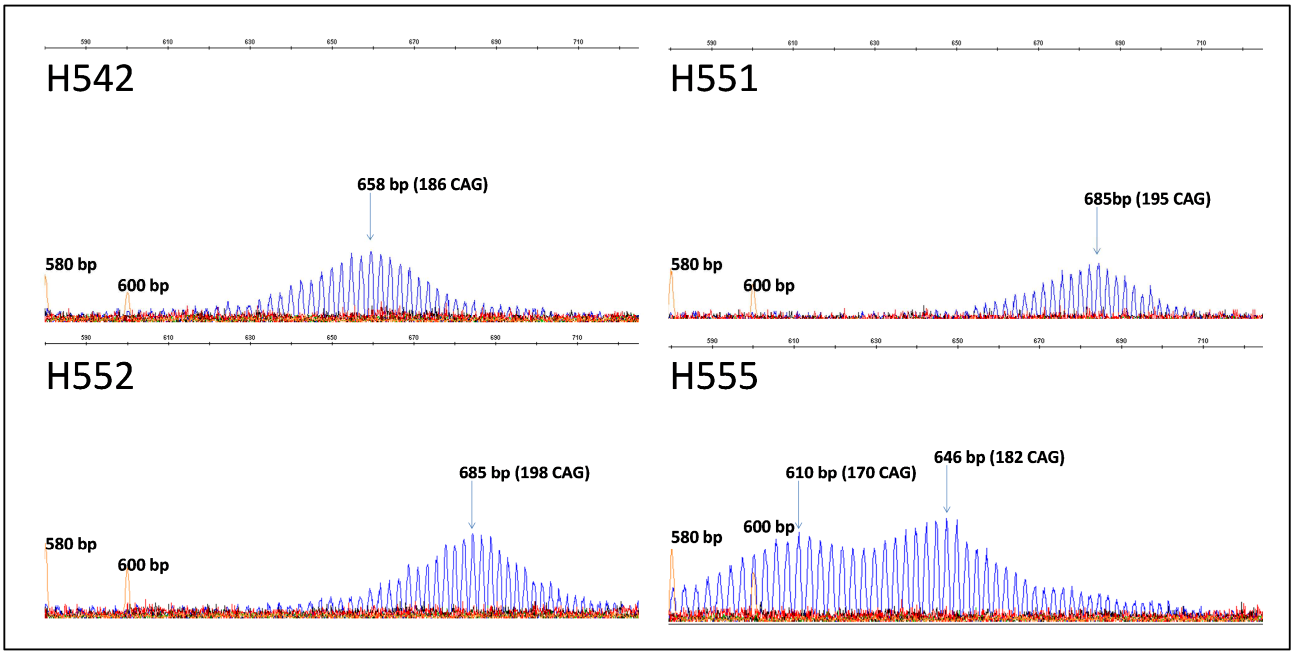

Metformin

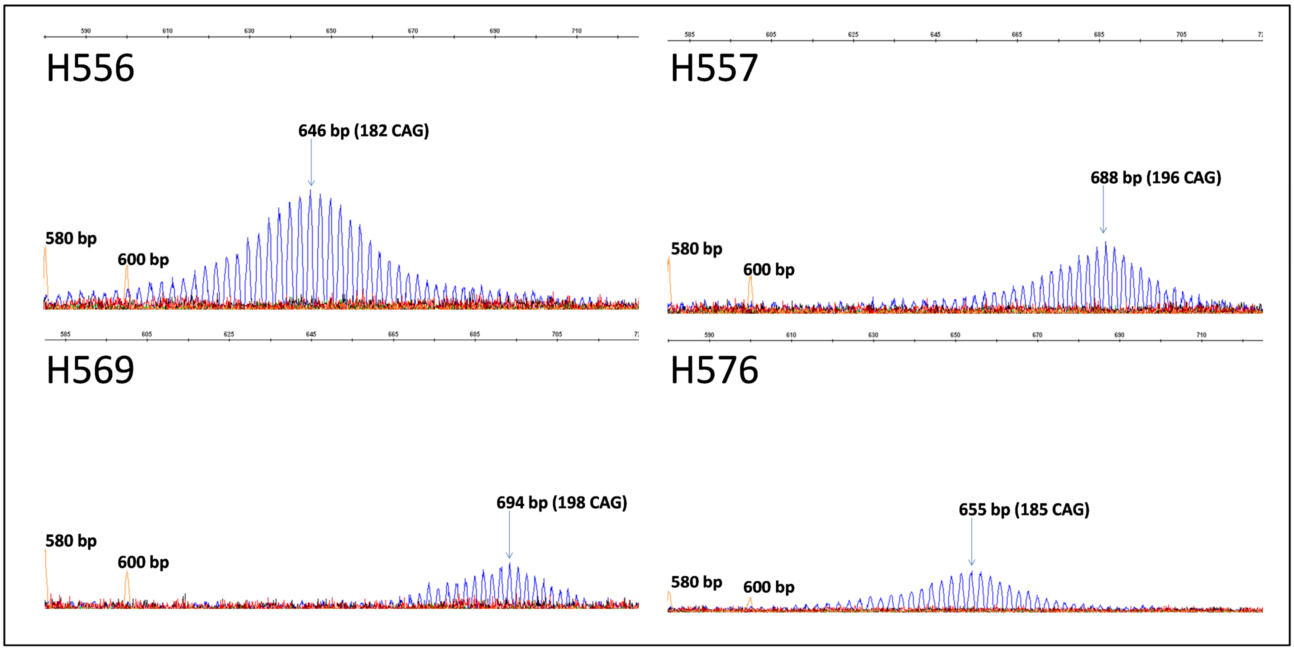

Fig. S5. Analysis of CAG expansions from cortex tissue from zQ175 mice. GeneMapper raw data of PCR products of the CAG expansions from control (n: 4) and metformin-treated (n: 4) six-month old mice do not show substantial difference in length. Peaks 580 and 600 bp corresponds to the last two band of the GeneScan 600 LIZ dye Size Standard v2.0. We point out the most likely average expansion of CAG triplets (blue arrow) and the corresponding number of CAG repeats. As in Fig S4, mouse H555 shows two different distributions.

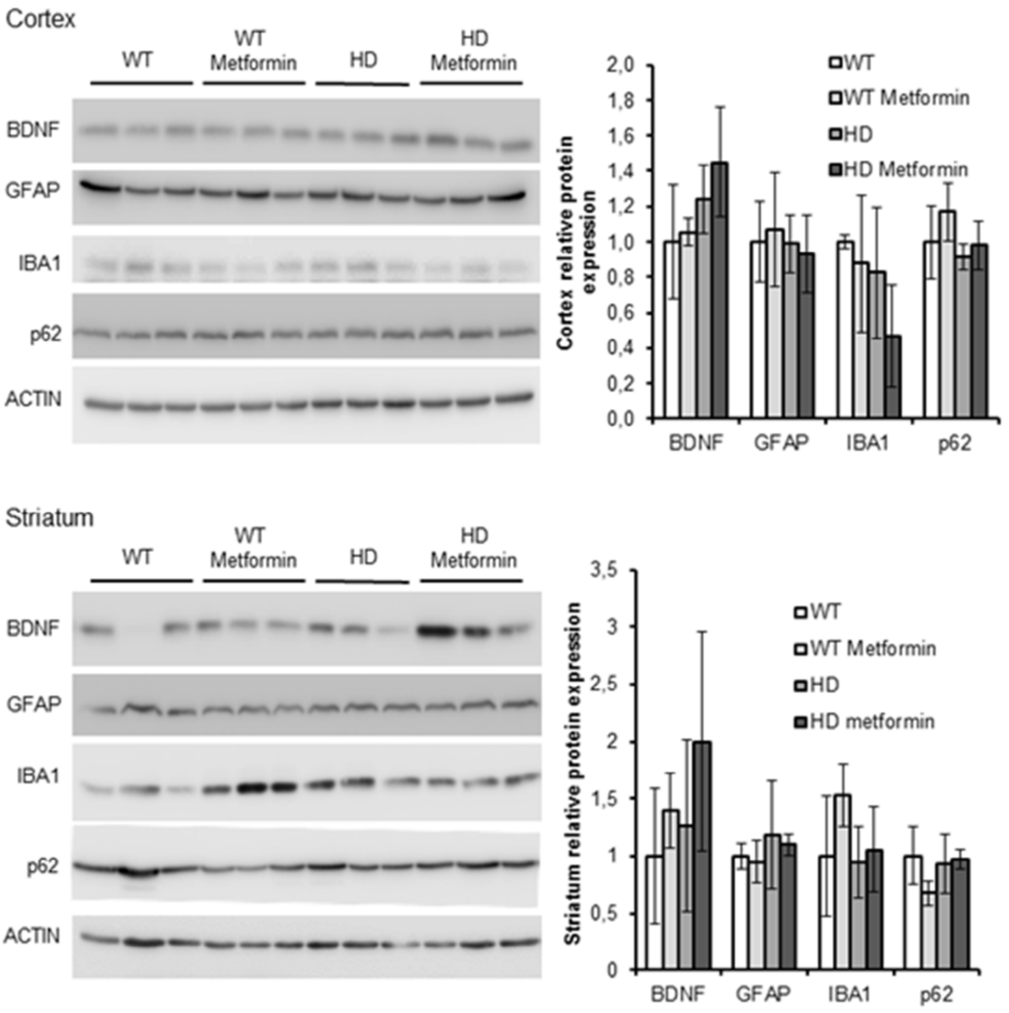

Fig S6. Molecular analysis of BDNF GFAP IBA1 and p62 by Western Blot of samples from cortex and striatum. 60 μg of cell extracts from cortex and striatum were analysed by SDS-PAGE and immunoblotting using the indicated antibodies. Samples from three independent mice were analysed. The intensity of the bands was related to the actin levels, and referred to the values obtained in untreated controls.

Table S1. Number of CAG triplets present in different samples from zQ175 heterozygous mice untreated (Control) or treated with metformin.

Mice	Treatment	Sample analysed	Mean Number of CAG triplets
H133	Control	Tail	189
H148	Control	Tail	189
H542	Control	Tail	187
H551	Control	Tail	193
H552	Control	Tail	194
H555	Control	Tail	170,182
H665	Control	Tail	197
H111	Metformin	Tail	188
H117	Metformin	Tail	184
H118	Metformin	Tail	182
H129	Metformin	Tail	189
H131	Metformin	Tail	188
H557	Metformin	Tail	195
H567	Metformin	Tail	186
H569	Metformin	Tail	198
H576	Metformin	Tail	184
H542	Control	Cortex	186
H551	Control	Cortex	195
H552	Control	Cortex	195
H555	Control	Cortex	170,182
H556	Metformin	Cortex	182
H557	Metformin	Cortex	196
H569	Metformin	Cortex	198
H576	Metformin	Cortex	185

Similar numbers of CAG triplets were found in peripheral tissue (tail) from control or metformin-treated animals. Similarly, no differences in CAG triples were found in the brain tissue (cortex) of control or metformin mice.
